# Metabolic fingerprints of *Serratia liquefaciens* under simulated Martian conditions using Biolog GN2 microarrays

**DOI:** 10.1038/s41598-018-33856-3

**Published:** 2018-10-24

**Authors:** Petra Schwendner, Andrew C. Schuerger

**Affiliations:** University of Florida, 505 Odyssey Way, Space Life Sciences Lab, Exploration Park, Merritt Island, FL 32953 USA

## Abstract

Microorganisms growing at atmospheric pressures of 0.7 kPa may have a significant impact on the search for life on Mars. Data on their nutrient requirements in a simulated Martian environment are required to ascertain both the potential risk of forward contamination and the potential of past or present habitability of Mars. *Serratia liquefaciens* can grow at concomitant conditions of low pressure, low temperature, and anoxic atmosphere. Changes in the metabolic fingerprint of *S. liquefaciens* grown under varying physical conditions including diverse atmospheric pressures (0.7 kPa to 101.3 kPa), temperatures (30 °C or 0 °C), and atmospheric gas compositions (Earth or CO_2_) were investigated using Biolog GN2 assays. Distinct patterns for each condition were observed. Above 10 kPa *S. liquefaciens* performed similar to Earth-normal pressure conditions (101.3 kPa) whereas below 10 kPa shifts in metabolic patterns were observed. The differences indicated a physiological alteration in which *S. liquefaciens* lost its ability to metabolize the majority of the provided carbon sources at 0.7 kPa with a significant decrease in the oxidation of amino acids. By measuring the physiological responses to different carbon sources we were able to identify nutritional constraints that support cellular replication under simulated shallow Mars subsurface conditions.

## Introduction

Identifying and protecting habitable zones (HZ) on Mars requires knowledge on the geochemical, environmental, and biological factors that may contribute to the colonization of HZ sites by hitchhiking spacecraft microorganisms^1,2^. In addition, characterizing the limits of terrestrial microbial life under realistic simulations of the Martian surface will inform the search for extant Martian life (if present) in plausible HZ terrains. Both efforts require data on the complex interactions of microbial metabolisms and the Martian environments being explored.

One constraint for robotic or crewed missions to Mars will be the question of whether Earth microorganisms have inherent abilities to survive, grow, and adapt to surface conditions on current-day Mars. One of the most challenging factors on Mars is low atmospheric pressure that ranges from 0.2 to 1 kPa, and equals about 1% of Earth sea-level pressure. Apart from the hypobaric conditions, microbial survival and growth also must cope with low water activities (a_w_) in soils, low temperatures, the presence of high salt concentrations and oxidizing compounds, low osmotic potentials in the regolith, high pCO_2_ and low pO_2_ atmospheres, as well as high doses of UV and ionizing radiation^3–5^. Recent studies have made progress in demonstrating microbial replication under a subset of the harsh conditions found on Mars by investigating whether bacteria can grow under hypobaric (close to 0.7 kPa), low temperature (near 0 °C for stable liquid water), and CO_2_-enriched anoxic conditions (henceforth called low-PTA conditions) similar to the Martian surface. Growth of 29 bacterial species under low-PTA conditions^5–7^ reveals active metabolism and growth at pressures encountered on the Martian surface, and consequently demonstrates the potential that some spacecraft microorganisms may be capable of colonizing hydrated terrains on Mars.

*Serratia liquefaciens* was one of the first bacteria shown capable of growth under low-PTA conditions^5^. *Serratia liquefaciens*, in the family Enterobacteriaceae, is a Gram-negative, motile, facultative anaerobic bacterium, and an ecological generalist. Its habitats include a variety of niches such as surface waters, soils, and the digestive tracts of rodents, plants, insects, fish, and humans^8^. Furthermore, *Serratia spp*. have been recovered from spacecraft assembly facilities^9,10^, Russian spacecraft^11^ and the International Space Station^12,13^ (ISS) which makes them plausible candidates to hitchhike on spacecraft to Mars. Experiments testing eight *Serratia* type strains confirmed the growth of *S. ficaria*, *S. fonticola*, *S. grimesii*, *S. liquefaciens*, *S. plymuthica*, and *S. quinivorans*, whereas *S. marcescens* and *S. rubidaea* failed to grow under low-PTA conditions^14^.

Low-PTA conditions represent a set of selective stressors which may introduce so far unknown alterations to cells rendering them unable to carry out normal metabolism and growth, or that may be lethal. Very little data exists on how microbial metabolism is affected by exposure to low-PTA conditions found on Mars. For example, it is unknown whether the same metabolic pathways (e.g., utilization of amino acids versus sugars) are active under low-PTA compared to Earth sea-level conditions.

Obtaining data on microbial metabolism and growth under simulated Martin conditions near 0.7 kPa is critical for modelling the potential risks of forward contamination in HZ regions on Mars^2,15,16^. In order to fill this knowledge gap we investigated carbon source utilization by *S. liquefaciens* using the Biolog GN2 assay system (Biolog, inc. Hayward, CA). The Biolog system was developed for bacterial identification based on species-specific metabolic fingerprints using the differential metabolism of 95 carbon sources^17^. Once a carbon substrate is metabolized (i.e., a substrate is oxidized) the colourless tetrazolium redox dye is reduced and forms a purple precipitate. The use of the tetrazolium reduction method allows for the quantification of differential substrate consumption providing estimates on the rates of metabolism on numerous organics under diverse conditions.

The objectives of the current study were to characterize carbon source utilization by *S. liquefaciens* under low-PTA conditions in order to (1) identify the effects of low pressure on metabolism, and (2) define unique organic substrates that are preferred by *S. liquefaciens* for metabolic activity and growth under simulated Mars surface conditions. Positive results from the latter objective were required to design ongoing complex simulations of microbial growth and adaptation to analogue soils and diverse environmental conditions on Mars (e.g., divergent a_w_, redox couples, and *in situ* organics).

## Results

Metabolic activity and substrate richness (number of positive wells) of *Serratia liquefaciens* cells were evaluated under different pressures, temperatures, and gas compositions in which one set of conditions simulated a Martian surface environment (i.e., low-PTA condition). Biolog GN2 microarray plates were inoculated to simultaneously test a wide variety of 95 carbon substrates. The assays were conducted in hypobaric systems capable of maintaining pressures between 0.7 kPa and 101.3 kPa (+/−0.1 kPa). The Biolog GN2 plate includes two alcohols, six amines, 20 amino acids, four aromatic substances, one brominated substrate, 28 carbohydrates, 24 carboxylic acids, two esters, three phosphorylated substrates, five polymers and one control well containing only water^17,18^. Initially, a series of preliminary tests confirmed that the GN2 plates with 10 mM PO_4_ buffer alone as the carrier fluid (i.e., without bacteria) remained free of colour-shifts for up to 60 days at 0.7 kPa, 0 °C, and CO_2_-enriched atmosphere. Consequently, any colour changes measured were interpreted to be the result of microbial metabolism and not the spontaneous reduction of the tetrazolium dye.

### Exp-1: Responses to various carbon sources in different atmospheric pressure conditions

In Exp-1, the temperature was held constant at 30 °C in an Earth-normal atmosphere of 78% pN_2_ and 21% pO_2_ present in the laboratory, and only pressure was modified to 2.5, 5.0, 10.0, 20.0, or 101.3 kPa (Earth-normal pressure). The Earth-normal atmosphere was created by allowing lab air to diffuse into the vacuum desiccators during the experiments. Due to the effects of low pressure on the evaporation rates of water, assays could not be conducted at pressures below 2.5 kPa, when incubated at 30 °C. Even at 2.5 kPa, the GN2 plates had to be partially rehydrated after 24 h of incubation during the 48-h assays. Rehydration was required to keep the nutrient concentrations and osmotic effects stable in each well throughout the experiment.

**Figure 1:**
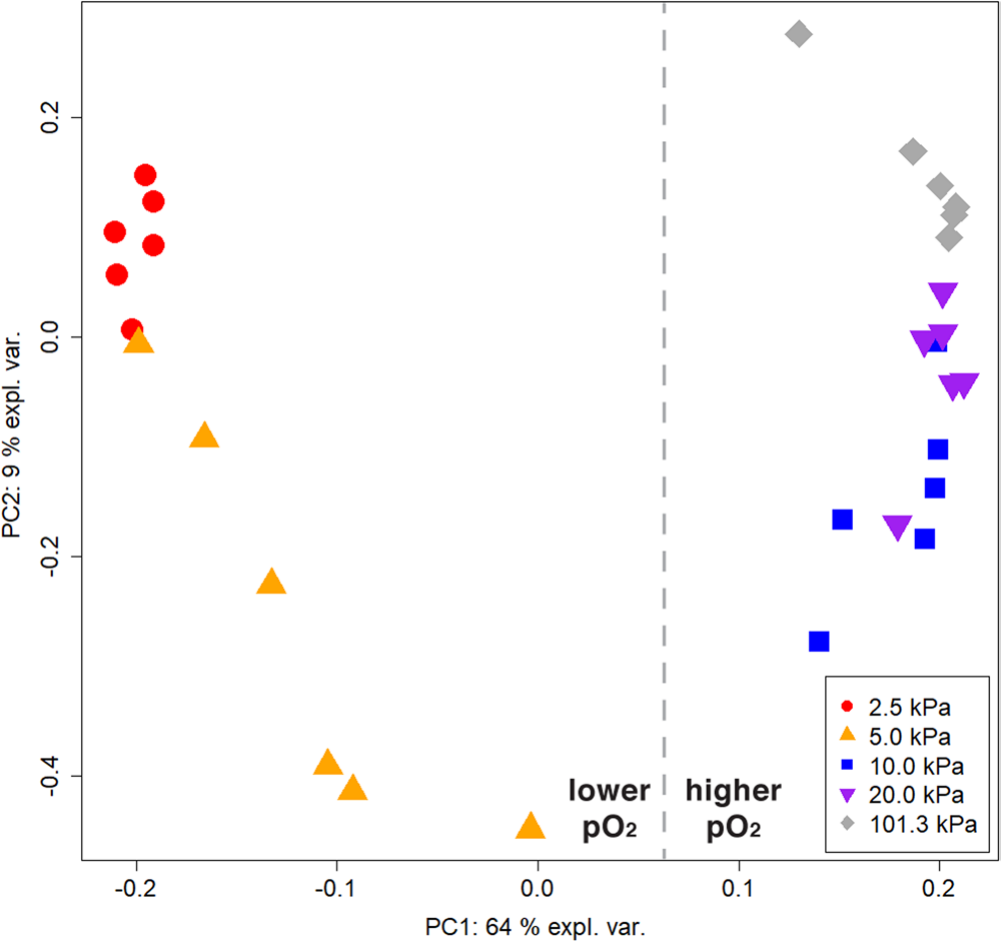
Substrate utilization patterns by *Serratia liquefaciens* under different pressures using Biolog GN2 microarrays. Ordination plot of metabolic fingerprints for cells grown at 30 °C; Earth-normal atmosphere; and pressures maintained at 2.5, 5.0, 10.0, 20.0, or 101.3 kPa (n = 6).

A principal component analysis (PCA) using the normalized absorbance values of the carbon sources as variables was performed to compare the carbon utilization profiles for the five tested pressures (Fig. 1). Principal component axis 1 accounted for 64% and PC axis 2 accounted for 9% of the total variance. The first PC separated the plates maintained at low pressures (2.5 and 5.0 kPa) from the plates incubated at higher pressures pointing towards different carbon utilization patterns amongst samples. Samples between 10.0 and 101.3 kPa grouped together indicating that pressures in this range only had a minor effect on carbon source utilization. There was no trend observed along the second principal component (PC2).

**Figure 2:**
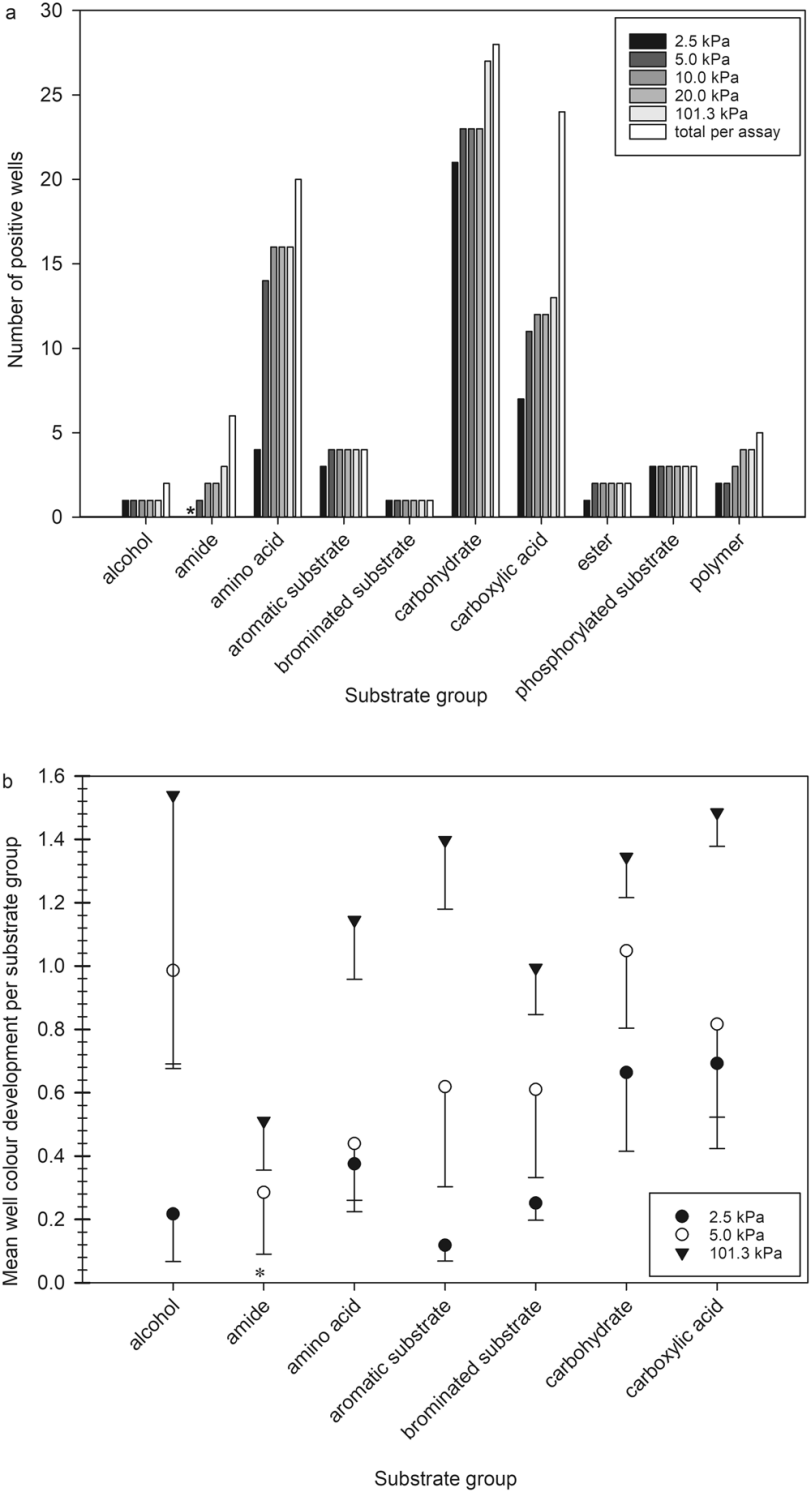
Overview of the metabolic fingerprints for *Serratia liquefaciens* cells grown under different atmospheric pressures using Biolog GN2 phenotypic microarrays. (**a**) The specific numbers of positive wells per substrate group are given for the following categories: alcohols, amides, amino acids, aromatic substrates, brominated substrates, carbohydrates, carboxylic acids, ester, phosphorylated substrates and polymers under the different pressure conditions (2.5, 5.0, 10.0, 20.0, 101.3 kPa) tested. (**b**) Comparison of OD readings between assays incubated at 2.5, 5.0, or 101.3 kPa. Error bars reflect standard deviations of the means between six independent replicates of the GN2 microarrays.

A summary of the overall carbon utilization patterns under different pressures is shown in Fig. 2a. A full list of substrates and their utilization can be found in Supplementary Table S1. Biolog GN2 plates kept at 2.5 and 5.0 kPa showed the smallest metabolic potential whereas samples incubated at Earth-normal pressure revealed the largest metabolic potential. While *S. liquefaciens* was able to utilize a total of 74 carbon sources at 101.3 kPa, the number of metabolized substrates decreased with decreasing atmospheric pressure to 41 at 2.5 kPa (Table 1). A similar trend was seen for the total well colour development (TWCD) with values ranging from 95 to 25 (i.e., for 101.3 to 2.5 kPa, respectively), and revealing only slight differences between 10.0 and 20.0 kPa (69 vs. 73). Thus, most changes occurred at pressures below 10.0 kPa. In addition, more than 50% of the positive reactions at 2.5 kPa showed very low OD values (i.e., <0.32) indicating low metabolic activity. Approximately half of the carbon sources used by *S. liquefaciens* at Earth-normal conditions supported metabolic activity at 2.5 kPa.

**Table 1:** Total well colour development (TWCD) values and number of carbon sources utilized by *Serratia liquefaciens* under different pressures.

Pressure (kPa)	TWCD	Carbon sources used
2.5	25.32 ± 9.27	41
5.0	50.44 ± 15.32	62
10.0	69.02 ± 13.47	69
20.0	73.70 ± 14.40	68
101.3	94.90 ± 12.01	74

The Biolog GN2 plates were inoculated with *S. liquefaciens* and incubated at 30 °C at the pressures indicated. Data are expressed as the sums for TWCD including standard deviations (n = 6).

**Figure 3:**
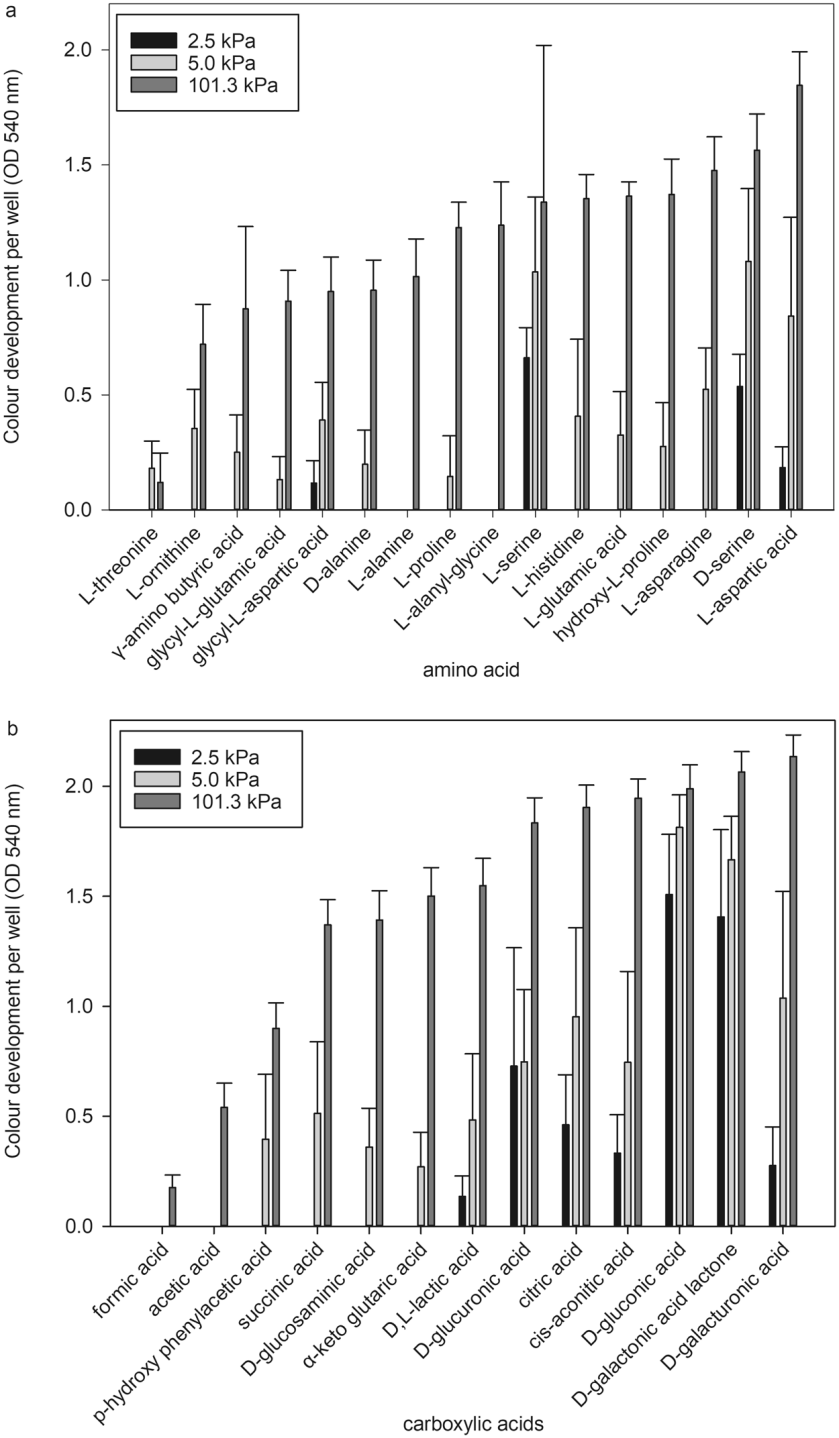
Detailed results of the organic compounds utilized by *Serratia liquefaciens* cells for amino acids and carboxylic acids tested at different pressures. (**a**) The OD colour development per well for each amino acid at 2.5, 5.0, or 101.3 kPa. (**b**) The OD colour development per well for each carboxylic acid at 2.5, 5, or 101.3 kPa. Error bars are standard deviations of the means between six independent replicates of the Biolog GN2 microarray assays.

Under Earth-normal conditions, *S. liquefaciens* was able to utilize almost all available carbohydrate (96%) sources, 80% of the amino acids, but only 58% of the carboxylic acids. The strongest reactions (individual well values above 1) were observed for the nine metabolites: β-methyl-D-glucoside, D-galactose, D-raffinose, sucrose, N-acetyl-D-glucosamine, D-trehalose, D-galactonic acid lactone, D-gluconic acid, and glucose-6-phosphate. The substrates exclusively used at 101.3 kPa were L-alaninamide, L-rhamnose, adonitol, xylitol, D-arabitol, and formic acid. In contrast, when *S. liquefaciens* was grown at 2.5 kPa, a major reduction in the ability to metabolize amino acids was observed reducing the overall utilization to 20%, although for most of the other tested chemical groups (e.g., carbohydrates, alcohols, carboxylic acids) only small or no changes were observed with regard to carbon utilization (Fig. 2a,b). The greatest pressure-dependent changes in substrate richness were observed for amino acids and carboxylic acids. At 2.5 kPa, *S. liquefaciens* was not able to metabolize the following 12 amino acids: L-threonine, L-ornithine, γ-amino butyric acid, glycyl-L-glutamic acid, D- and L-alanine, L-proline, L-alanyl-glycine, L-histidine, L-glutamic acid, hydroxyl-L-proline, and L-asparagine (Fig. 3a), and the six carboxylic acids: formic acid, acetic acid, p-hydroxy phenylacetic acid, succinic acid, D-glucosaminic acid and α-keto glutaric acid (Fig. 3b). The highest OD, i.e., greatest response, was observed for the amino acids L- and D-serine, and the carboxylic acids D-gluconic acid and D-galactonic acid lactone. At 5.0 kPa, *S. liquefaciens* was not able to utilize two amino acids (L-alanine and L-alanyl-glycine) and two carboxylic acids (formic acid and acetic acid).

### Exp-2: Metabolic fingerprints and the effect of incubation time for low-PTA conditions and controls

In a second series of experiments, the metabolic fingerprint for *S. liquefaciens* was investigated after incubation for 35, 42 and 49 days at: (1) low-PTA conditions, 0.7 kPa, 0 °C, CO_2_-enriched anoxic atmosphere, (2) Earth-control 1 (EC-1, see Methods) with Earth atmospheric pressure, 0 °C, CO_2_, (3) EC-2 with Earth atmospheric pressure, 0 °C, O_2_, (4) EC-3 with Earth atmospheric pressure, 30 °C, CO_2_-enriched anoxic atmosphere, and (5) EC-4 with Earth atmospheric pressure, 30 °C, O_2_. The specific carbon sources that are, or are not, utilized may indicate which metabolic pathways are able to function in hypobaric atmospheres like the Martian surface. After 35, 42, and 49 days of incubation OD readings were taken to determine whether incubation time had an effect on the metabolic fingerprint. After the completion of the three different 49-d assays at 0 °C, the plates were further incubated for 48 h at 101.3 kPa, 30 °C under an Earth-normal pO_2_ atmosphere. The extra 48 h incubations permitted us to evaluate if the cells in negative wells would return to normal metabolic functions as observed in optimal growth conditions.

**Figure 4:**
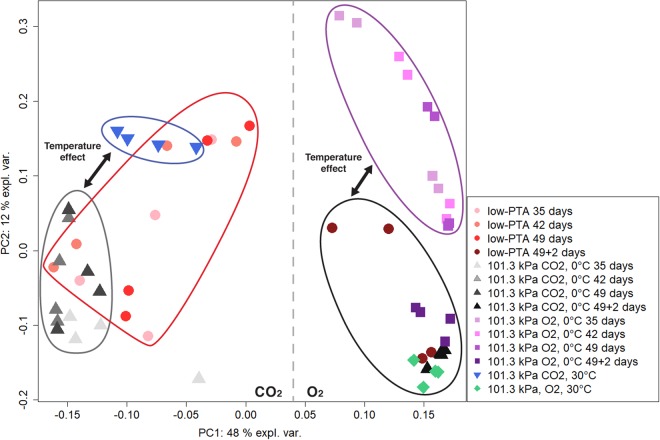
Overview of substrate utilization patterns in *Serratia liquefaciens* under low-PTA conditions and Earth controls at different incubation times. Ordination plot generated by principal component analysis of metabolic fingerprints at (1) low-PTA conditions of 0.7 kPa, 0 °C, and CO_2_-enriched anoxic atmosphere; (2) Earth atmospheric pressure, 0 °C, and CO_2_; (3) Earth atmospheric pressure, 0 °C, and lab-normal pO_2_; (4) Earth atmospheric pressure, 30 °C, and CO_2_-enriched anoxic atmosphere; and (5) Earth atmospheric pressure, 30 °C, lab-normal pO_2_. Data were taken on 35, 42, or 49 days. The 49 + 2 days treatments refer to Biolog GN2 plates incubated at 30 °C, Earth-normal pressures, and lab-normal atmospheres for an additional 2 d after termination of incubation conditions in (1) through (3) above.

To identify the effects of the treatments (14 data sets, n = 4) on the metabolic fingerprints and whether there were distinct changes in substrate richness, PCA was performed. In the ordination of Fig. 4, the first two principal components accounted for 60% of the overall variance. The multivariate analysis revealed four clusters containing only data points from the conditions (EC-2) Earth atmospheric pressure, 0 °C, CO_2_, (EC-3) Earth atmospheric pressure, 0 °C, O_2_, (EC-4) Earth atmospheric pressure, 30 °C, CO_2_-enriched anoxic atmosphere, and (EC-5) Earth atmospheric pressure, 30 °C, O_2_, respectively. The assays from the low-PTA condition, which are spread among the samples from conditions EC-1 and EC-3, were the only exceptions. As expected all 49 + 2 d samples grouped with condition (EC-4) with some outliers from the low-PTA conditions.

Upon return of the plates from low-PTA conditions to 101.3 kPa at 30 °C, the cells in most substrate wells (i.e., 64 out of 74) resumed growth and the Earth-normal metabolic fingerprint pattern was observed with slight variations (see Table 2). Colour development in 10 wells failed when the Biolog plates were returned to optimal growth conditions. The reduced number of metabolized carbon sources demonstrated that cells of *S. liquefaciens* incubated for long periods at 0.7 kPa were not able to fully return to normal metabolic activity under optimal growth conditions. The results might indicate that some cellular machinery for carbon utilization was inhibited or turned off after long exposures to low pressures. In addition, a separation of the data set along PC1 accounting for 48% of the variance was identified that differentiated samples grown under O_2_ from those grown under CO_2_-enriched anoxic conditions (Fig. 4). Furthermore, a distinct effect of temperature on the metabolic fingerprint was observed when comparing samples grown in an aerobic environment at 30 °C (Fig. 4, green diamonds) compared to samples grown at 0 °C (Fig. 4, purple squares) but at the same atmospheric pressure. A similar effect was observed for samples grown in the CO_2_-enriched environment and Earth-normal pressure at 30 °C (Fig. 4, blue triangles) versus 0 °C (Fig. 4, grey triangles).

**Table 2:** Total well colour development (TWCD) values and numbers of carbon sources utilized by *Serratia liquefaciens* under different pressures between 35 and 49 d.

Condition	Days of incubation	TWCD	Carbon sources used
Low-PTA	35	4.24 ± 1.35	16
	42	4.67 ± 2.47	21
	49	7.49 ± 2.10	24
	49 + 2	48.66 ± 20.99	64
101.3 kPa, CO_2_, 0 °C	35	1.80 ± 0.22	5
	42	2.92 ± 0.40	8
	49	4.32 ± 1.14	13
	49 + 2	74.94 ± 8.67	72
101.3 kPa, O_2_, 0 °C	35	24.39 ± 6.53	53
	42	28.53 ± 4.90	54
	49	32.59 ± 4.25	55
	49 + 2	79.09 ± 17.62	66
101.3 kPa, CO_2_, 30 °C	2	32.95 ± 2.63	46
101.3 kPa, O_2_, 30 °C	2	90.2 ± 8.18	74

After the experiments were terminated, the GN2 plates were incubated for an additional 48 h at 30 °C (i.e., the 49 + 2 treatments). The GN2 plates were inoculated with *S. liquefaciens*. Data are expressed as the sums (for TWCD) including standard deviations (n = 4).

**Figure 5:**
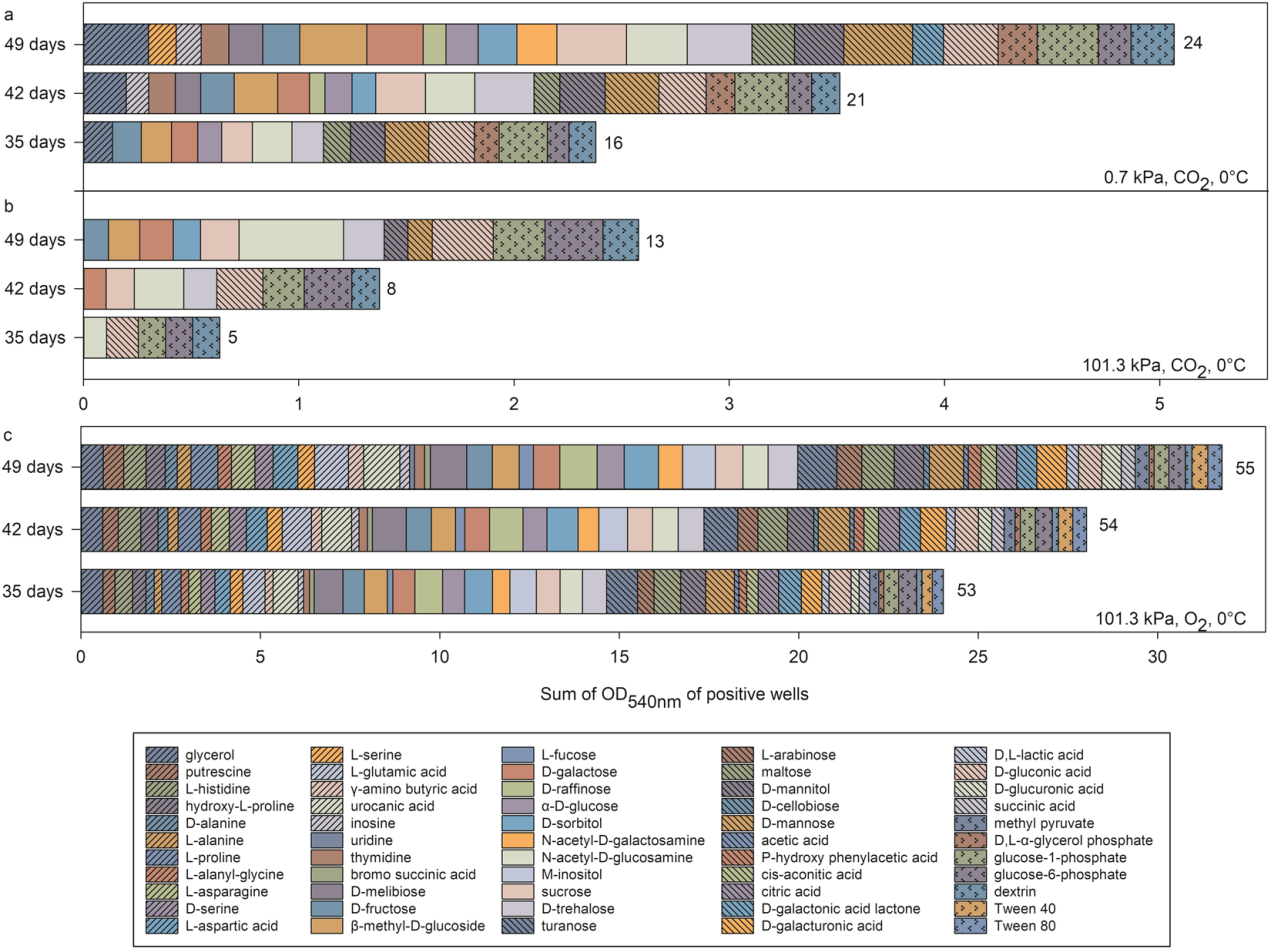
Comparisons of substrate utilization patterns for *Serratia liquefaciens* cells grown at 0 °C under 0.7 or 101.3 kPa and under pO_2_ or pCO_2_. The OD readings were taken after 35, 42, and 49 d of incubation. The number at the end of each horizontal bar indicates substrate richness. The assay plates were incubated at (**a**) 0.7 kPa, CO_2_ atmosphere, and 0 °C; (**b**) 101.3 kPa, CO_2_ atmosphere, and 0 °C; or (**c**) 101.3 kPa, CO_2_ atmosphere, and 0 °C. The OD data represent average values of n = 3 replicates.

At 0 °C, colour development rates in many wells were delayed, and thus, presumably the growth or metabolic rates were retarded (Fig. 5 and TWCD values in Table 2). We observed a lag period of 28 d, after which, the colour development increased gradually until the final measurements were collected at 49 d. The lag period was likely due to slower growth rates at 0 °C compared to 30 °C, and that specific cell densities (i.e., ~10^8^ cells/ml) had to be achieved before colour development was measureable^17^. The time course for colour development of the positive substrates under the various tested conditions is shown in Fig. 5. The results indicated that the rates of substrate oxidation were not linear throughout the incubation period. Consequently, the numbers of positive wells increased with incubation time which means that readings at earlier days led to an underestimation of the overall potential substrate richness for *S. liquefaciens*. For example, under low-PTA conditions at 0.7 kPa, substrate richness increased from 16 to 24 between days 35 and 49. In addition, metabolic activity was suppressed at all three sample dates for cells grown under CO_2_ at 0 °C atmospheres compared to low-PTA conditions, suggesting that low-PTA conditions may have “relaxed” a metabolic bottle-neck for many organics present in the Earth-normal conditions of 101.3 kPa, CO_2_, and 0 °C.

The highest OD values, hence the highest metabolic activities, were observed for glycerol, β-methyl-D-glucose, sucrose, D-mannose, n-acetyl-glucosamine (OD ~ 0.3) when cells were grown at low-PTA conditions; n-acetyl-glucosamine, D-gluconic acid, glucose-6-phosphate (OD = 0.48–0.26) when cells were grown at Earth atmospheric pressure, 0 °C, CO_2_; and D-melibiose, urocanic acid, D-raffinose, and turanose (OD > 1) when cells were grown in Earth atmospheric pressure, 0 °C, and O_2_ (Fig. 5). The observed differences suggest that different metabolic pathways were active under the diverse incubation conditions tested (Fig. 5, Supplementary Table S2).

After 49 d, the TWCD values were 7.49 (+/−2.10) at 0.7 kPa compared to 4.32 (+/−1.14) at 101.3 kPa, CO_2_, 0 °C (Table 2); a second example that low-PTA conditions seemed to relax a metabolic bottle-neck for cells grown under low temperature and CO_2_-enriched anoxic conditions. Accordingly, *S. liquefaciens* was able to use a total of 24 carbon sources at 0.7 kPa compared to only 13 at 101.3 kPa, CO_2_, 0 °C. In the latter case, nine carbohydrates, two phosphorylated substrates, one polymer, and one carboxylic acid were utilized. At 0.7 kPa *S. liquefaciens* used an additional six carbohydrates, two amino acids, one carbohydrate, one ester, one alcohol, one carboxylic acid, and two aromatic compounds. A similar trend was observed when comparing the two Earth controls (EC-3 and EC-4) incubated at 30 °C. A higher TWCD and a higher number of carbon sources were used in the aerobic environment (Table 2).

**Figure 6:**
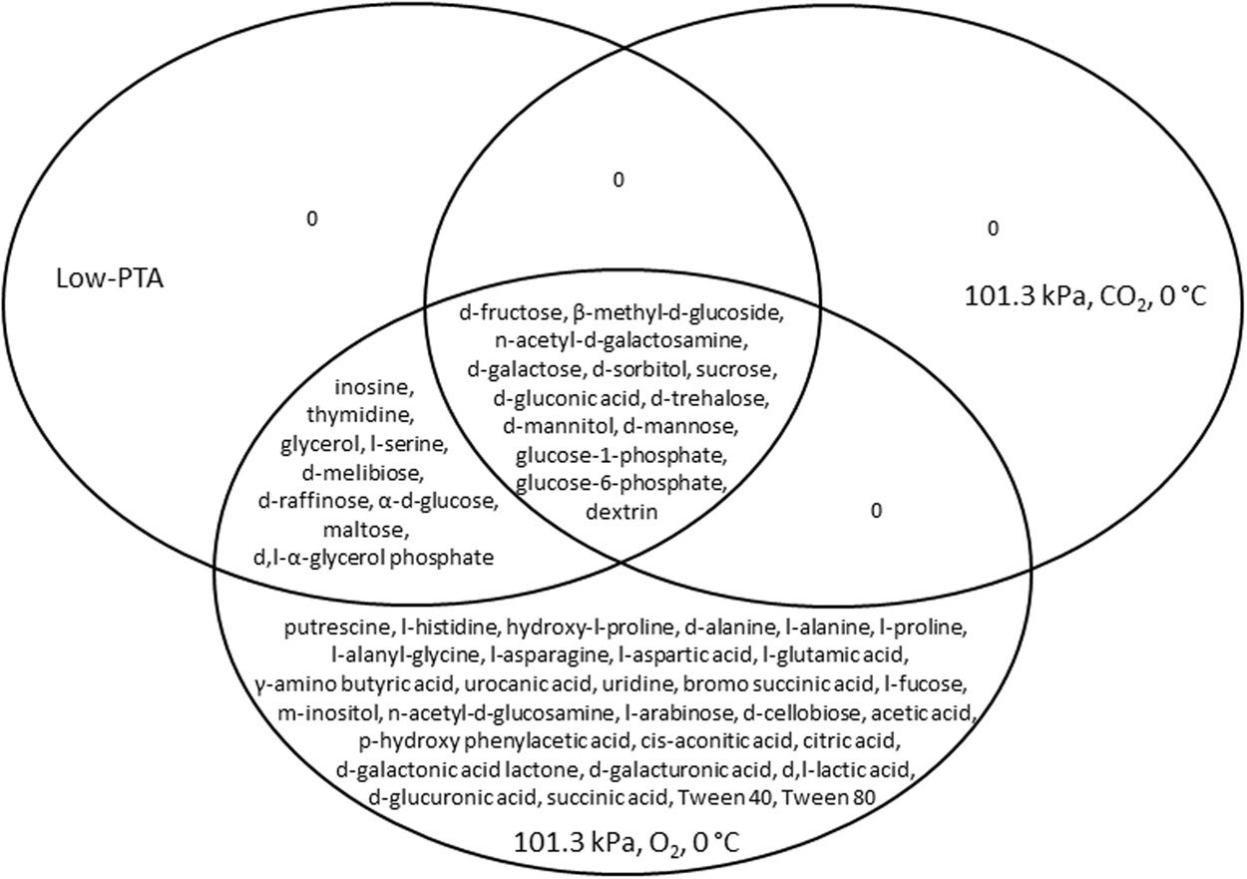
Venn diagram of the carbon sources utilized by *Serratia liquefaciens* after 49 d of incubation under three different growth conditions. Comparison of data from GN2 assay plates (n = 3) that were incubated at (1) low-PTA conditions at 0.7 kPa, CO_2_ atmosphere, and 0 °C; (2) 101.3 kPa, CO_2_ atmosphere, and 0 °C; or (3)101.3 kPa, CO_2_ atmosphere, and 0 °C.

Comparison of metabolic fingerprints between O_2_ and CO_2_ atmospheres incubated at Earth-normal pressures and 30 °C demonstrated that the gas composition had an effect (Fig. 4). *S. liquefaciens* likewise used 46 substrates and an additional 28 substrates were metabolized when grown in an aerobic environment (Table 2). Comparison of carbon utilization of plates incubated at 0 °C and 30 °C, Earth normal pressure and O_2_ atmosphere highlighted the effects of temperature among the two temperatures tested (Fig. 4). *S. liquefaciens* likewise used 55 substrates at both temperatures tested, despite the fact that the metabolic rate was slower at 0 °C compared to 30 °C. Furthermore, *S. liquefaciens* was able to utilize an additional 19 carbon sources when grown at 30 °C (Table 2). Despite the observed differences, we were able to identify a core metabolic fingerprint for *S. liquefaciens* cells grown under all conditions (Fig. 6). The catabolism of 17% of the 95 organics, consisting mainly of carbohydrates, was not affected by either temperature, pressure, or gas composition. None of the tested substrates were exclusively utilized at either low-PTA, or under 101.3 kPa, CO_2_ and 0 °C conditions (Fig. 6).

Under optimal growth conditions (i.e., EC-4 at 101.3 kPa, O_2_, and 30 °C), *S. liquefaciens* metabolized the majority (n = 74) of the carbon sources. In contrast and under all of the tested conditions, *S. liquefaciens* was unable to utilize the following 18 substrates: 2,3-butanediol (alcohol), i-erythritol (carbohydrate); the amides succinamic acid, 2-aminoethanol, phenylethylamine; the amino acids L-leucine, L-phenylalanine, L-pyroglutamic acid; the carboxylic acids α-hydroxy butyric acid, α-keto butyric acid, β-hydroxy butyric acid, γ-hydroxy butyric acid, itaconic acid, malonic acid, propionic acid, quinic acid, sebacic acid; and the polymer α-cyclodextrin.

## Discussion

If Earth bacteria can be shown to possess the metabolic range to grow in 0.7 kPa, 0 °C, and CO_2_ anoxic atmosphere this would increase the possibility that the Martian environment is habitable to Earth-like life, and may indicate that specific niches might be candidate locations to search for an extant Mars microbiota. While low pressure environments exist in high-altitude environments on Earth, or in extraterrestrial settings like the surface of Mars, hypobaric conditions cannot be found on Earth’s surface. Despite these ecological constraints, Earth bacteria have been identified with the ability to grow in low-pressure conditions^5–7^. One model organism for hypobaric studies is *S. liquefaciens* exhibiting a wide temperature growth range and versatility to tolerate various extremes, such as growth without oxygen or at hypobaric conditions. Thus, by fully characterizing the limits of growth under simulated Martian conditions, we can more effectively identify and protect niches on Mars that might harbour an extant microbial community. While many studies have been conducted to test microbial survivability under Martian conditions, with a focus on radiation and/or desiccation effects under Martian atmospheric gas composition^19–24^, only a few studies have looked into the ability of microorganisms to grow in low-pressure environmental settings^5–7,25,26^. These hypobaric studies revealed that the growth of the majority of microorganisms was inhibited indicating that low pressure may be a selective stress which constitutes a barrier for potentially growing on Mars. Consequently, assessing the metabolic responses to simulated Martian conditions and characterizing their carbon source utilization patterns are of critical importance when evaluating the risks for the forward contamination of HZ terrains.

To investigate the effects of low pressure under simulated Martian conditions on the metabolic fingerprint of *S. liquefaciens*, Biolog GN2 microarrays were used which have been successfully tested under low pO_2_ and low temperature conditions^27^. The microarrays are based on the measurements of redox potential changes in accordance with purple-colour development of a redox sensitive dye which has been proven very useful for qualitative and quantitative determination of microbial metabolism under aerobic and most anaerobic conditions^28–30^. However, the Biolog GN2 microarrays have not yet been tested under hypobaric conditions. Our experiments confirmed that the Biolog GN2 microarray system can be used to identify sole carbon sources for *S. liquefaciens* metabolism under simulated Martian conditions at 0.7 kPa.

Under optimal growth conditions (EC-4; 30 °C, pO_2_ (21%), Earth-normal pressure) *S. liquefaciens* displayed a high versatility in carbon utilization with the ability to metabolize 74 out of 95 substrates including alcohols, amines, amino acids, aromatic compounds, carboxylic acids, carbohydrates, esters, polymers, phosphorylated, and brominated substances. *Serratia liquefaciens* is a facultatively anaerobic heterotroph capable of growth under low pO_2_ and able to utilize a wide diversity of organics for metabolism. Isolates have been recovered from numerous ecological niches including the drinking water onboard the ISS^12^; plant rhizospheres, fruits, grasses, and vegetables^31–33^; processed foods^34,35^; meats^34,36^; or as opportunistic pathogens in fish^37^ and the human bloodstream^38^.

Apart from determining the substrate richness at optimal growth conditions, one of the main objectives investigated in Exp-1 was whether a single stressor such as pressure would alter the metabolic fingerprint of *S. liquefaciens*. Pressures from 2.5–101.3 kPa (at 30 °C) were tested to determine the physical limits in which low atmospheric pressures would begin to alter cellular metabolism. Compared to optimal growth conditions, the TWCD decreased at low pressures. The Biolog GN2 protocol allows the investigation of both metabolism alone, when starting cell densities are ≥10^8^ cells/well, or cell growth plus metabolism, when starting cell densities are ~10^5^ cells/well^17,39^. Because the GN2 assays described here were started with low cell densities at ~2 × 10^5^ cells per well, results indicate lower metabolic activities and slower growth rates for *S. liquefaciens* cells incubated at low pressures.

The hypobaric effect of suppressing metabolism was confirmed by PCA (Fig. 1) separating the data into two groups with one cluster representing samples incubated at higher pO_2_ and the other representing samples incubated at lower pO_2_. Above 10.0 kPa, *S. liquefaciens* performed similarly to Earth-normal conditions whereas below 10.0 kPa a shift in the metabolic fingerprint and a reduction in growth rates were observed. The hypobaric effects of suppressing growth described here were similar to those observed for seven *Bacillus* spp. in which lowering atmospheric pressure led to reduction and then cessation of growth below 5 kPa^7^. Similarly, *Deinococcus radiodurans* R1 was found to grow at low pressures down to only 10.0 kPa^4,5^. The differences between the low pressures at 5 kPa or below and all higher pressures also may have been due to the effects of low pO_2_ (at 2.5 and 5 kPa) versus higher pO_2_ (10, 20, or 101.3 kPa), but the role of pO_2_ on carbon utilization was not independently studied here. These data indicate that pressures below 10.0 kPa induce a selective stress that can be described as a physical barrier for limiting growth in the majority of bacteria, while pressures above 10.0 kPa altered microbial activity to only a minor extent.

In Exp-2, a long lag phase was discovered when cultures were grown at 0 °C, and thus, quantitative microarray plate readings were started at 35 d. Based on early work in which colonies of *S. liquefaciens* on TSA agar surfaces occurred only after 10–14 days of incubation under low-PTA conditions^5,14^, the observed delay was anticipated. Haack *et al*.^40^ showed that colour development rates in wells are not always linear, and that substrate oxidation profiles follow similar sigmoidal functions as observed for bacterial growth curves. In addition, substrate oxidation profiles are not always directly linked with bacterial growth^40^.

When comparing all samples from Exp-2, we found that low-PTA conditions had a cumulative impact, as shown by PCA (Fig. 4), indicating that the metabolic fingerprints were affected by pO_2_, temperature, and pressure. The lowest rates of metabolism and carbon source utilization occurred when cells were incubated under 101.3 kPa, 0 °C, and CO_2_-enriched anoxic atmosphere. These results are consistent with previous observations that demonstrated *S. liquefaciens*^5^ and *Carnobacterium* sp.^6^ grew better under low-PTA conditions compared to 101.3 kPa at 0 °C and CO_2_-enriched anoxic atmospheres. The mechanism that increases growth rates of *S. liquefaciens* cells under low-PTA conditions compared to growth at 101.3 kPa, 0 °C, and CO_2_-enriched anoxic atmospheres is unknown.

Under low-PTA conditions *S. liquefaciens* favoured carbohydrates for metabolism over most other organic classes. In contrast, most of the amino acids used by *S. liquefaciens* under Earth-lab conditions of 101.3 kPa, 30 °C, and O_2_ were not metabolized by cells incubated at, or below, 2.5 kPa, regardless of the other conditions tested. For example, L-aspartic acid, glycyl-aspartic acids, D-serine, and L-serine were utilized at 2.5 kPa, but only L-serine was utilized under low-PTA conditions. The ability to metabolize carbohydrates and the inability to metabolize most amino acids under low-PTA conditions at 0.7 kPa are consistent with a transcriptomics study of *S. liquefaciens* conducted at the same low-PTA conditions as tested here^41^ in which the up-regulation of numerous genes in carbohydrate metabolic pathways and the down-regulation of various genes in amino acid pathways were demonstrated.

In the following sections, several hypotheses are discussed that may explain how the different metabolic rates of carbohydrates and amino acids are altered at low-PTA conditions; leading to the conclusion that the different metabolic rates are caused by the interplay of several factors rather than the effect of just one factor. At lower pressures and in CO_2_ atmosphere, the pO_2_ is significantly lower compared to the conditions under Earth-normal atmosphere. This leads to the induction of an anaerobic metabolism using carbohydrates for fermentation and anaerobic respiration processes. Results are consistent with the conclusion that *S. liquefaciens* favours carbohydrates over most other chemical classes under low-PTA conditions.

Incubation at temperatures below the optimum growth conditions has impacts not only on cell integrity and membrane fluidity but also on the metabolic rates due to altered enzyme kinetics. Thus, lower TWCD values for plates grown at 0 °C were expected, which is consistent with low temperatures having strong effects on amino acid and carbohydrate metabolisms^42,43^. However, in our study when comparing the metabolic fingerprints at 101.3 kPa, O_2_, 0 °C, with those at 30 °C, only small changes in the substrate utilization of amino acids were detected. Therefore, changes in the amino acid utilization patterns at low-PTA conditions observed here can be attributed to lower pressures.

Due to the paucity of data on bacterial metabolism under hypobaric conditions, a definitive explanation for why *S. liquefaciens* prefers carbohydrates over amino acids under low-PTA conditions is lacking. However, another approach to develop potential insights on microbial metabolism at low pressures is to compare the metabolisms of high-pressure tolerant bacteria (i.e., piezophiles) grown under their normal high-pressure environments (MPa range) with Earth-sea level pressures at 101.3 kPa (i.e., effectively a low-pressure environment for the piezophiles). For example, transcriptomes of *Photobacterium profundum* revealed an up regulation of genes involved in amino acid membrane transport and metabolism at 101.3 kPa (i.e., low pressure for *P. profundum*) compared to the optimal piezophilic environment at 28 MPa^44^. This upregulation was proposed as a strategy to compensate for the decreased activity of proteins and membrane functionality^45^.

Furthermore, membrane transporter systems are pressure sensitive with the majority of studies investigating the effects of high pressure. At 50 MPa, Paul and Morita^46^ reported up to a 90% inhibition for the transport of amino acids across membranes by *Vibrio sp*., which was suggested as the result of a conformational change of the membrane rather than the loss of function of the respiratory enzymes. In another example, Fajardo-Cavazos *et al*.^4^ found that low pressure led to changes in membrane composition when *Bacillus subtilis* was grown as 5.0 kPa compared to 101.3 kPa. In particular, while the ratio of unsaturated to saturated fatty acids decreased, the ratio of anteiso- to iso-fatty acids increased. The latter also has been implicated in microbial adaption to high pressure environments^47^. And finally, it is known that low temperature has a negative effect on membrane fluidity^48^. These observations suggest a mechanism in which low pressures alter membrane functionality by changing the permeability, fluidity, or protein movement. Furthermore, loss of membrane functionality might lead to interruptions in permeability, and therefore interact adversely with the uptake of certain classes of carbon sources. The exact role of low pressure and its potential effect on the membrane remains to be elucidated.

Apart from the influence that membrane permeability might have on amino acid uptake, the decreased utilization of amino acids at low pressures might be the result of physiological changes in cells. For example, *S. liquefaciens* cells possess flagella and are able to swarm^49^. Amino acids improve swarming capabilities in bacteria which require high demands for both organics and energy to synthesize and operate the flagella produced during swarming differentiation^50^. Under stressed conditions, like low pressures, *S. liquefaciens* cells may lose their motility and hence the demand for amino acids, and thus, prioritize vital functions that allow survival. A similar observation (e.g., a gradual decrease in cell motility) has been made for *Shewanella oneidensis* when grown at high pressure^51^.

## Conclusions

The search for life on Mars remains a key objective of the Mars Exploration Program^52^, and numerous studies have proposed methods for characterizing HZ’s in the shallow subsurface^2,3,53^. Furthermore, it is imperative to avoid forward contamination of Special Regions on Mars with surface vehicles in order to protect the pristine terrains being explored^2^. Results from the current project are thus useful for both mitigating the forward contamination of exploration sites and the search for life on Mars.

Many of the carbon sources utilized by *S. liquefaciens* under low-PTA conditions (e.g., aliphatic compounds, PAHs, sugars, aldehydes, carboxylic acids) have been reported in interplanetary dust particles (IDPs) or carbonaceous chondrites^54–56^. And recently, indigenous aromatic and aliphatic organics were detected in lacustrine mudstones in Gale Crater^57^. Results here suggest that *S. liquefaciens* may be able to grow on Mars if transferred to stable hydrated niches that contain indigenous or accreted IDP/chondritic organics similar to the organics used in the Biolog GN2 microarrays. Furthermore, previous work showed that *B. subtilis* and *Enterococcus faecalis*^58,59^ and *S. liquefaciens*^26,60^ can survive contact with Martian regolith indicating that Martian regolith may not be overtly biotoxic to many of the bacteria typically recovered from spacecraft surfaces.

The Biolog GN2 microarray protocols developed here could be used to quickly screen spacecraft microorganisms for growth under simulated Martian conditions while also focusing on the utilization of the organics likely present on Mars. If specific organics (e.g., glycerol, β-methyl-D-glucose, sucrose, D-mannose, N-acetyl-glucosamine used by *S. liquefaciens* at 0.7 kPa) are found to be all utilized by a wider range of hypobarophiles, then Martian terrains that are shown to possess such organics (i.e., during *in situ* robotic landers or rovers experiments) could be identified as potential habitats for terrestrial life. Consequently, these sites might pose potential risks for the forward contamination of specific terrains. For example, if individual organics are shown to be present on Mars, pre-launch planetary protection protocols for follow-on missions could be enhanced for surface vehicles to prevent the forward contamination of the revisited sites.

And lastly, the search for life on Mars requires us to not only *follow the water* but also to *follow the organics*. Future rover/lander payloads must be developed that consider the ecological conditions for microbial growth in Special Regions being explored in order to prioritize niches that have a multiplicity of conducive conditions in which a putative extant Mars microbiota might be present. Organics that are part of the *follow the water/organics* strategy, and can be used by a diversity of Earth microorganisms under simulated low-PTA conditions, will inform the search for life on Mars and avoid microbial conflicts between the Earth (on spacecraft) and Martian (if present) microbiota.

## Material and Methods

### Microbial Procedures

*Serratia liquefaciens* (ATCC 27592) cells were grown on full-strength trypticase soy agar (TSA; 40 g L^−1^) (Difco media, Fisher Scientific, Pittsburgh, PA, USA) for 24 h at 30 °C. Cells were harvested in 10 mM sodium-phosphate buffer (PO_4_ buffer; pH 7.2), diluted to yield approx. 2.0 × 10^6^ cells per mL (OD = 0.005–0.012 at 400 nm; average OD = 0.008), and stored at 24 °C for no more than 1 h before dispensing the cells into Biolog GN2 plates, as described below. Numbers of viable cells were estimated for each replicate by serially diluting inoculum through six, 10-fold dilutions and dispensing aliquots of each dilution into wells of a Most Probable Number (MPN) assay as described by Schuerger *et al*.^23^.

### Biolog inoculation and incubation protocols

Biolog GN2 microarrays (Biolog, Inc., Haywood, CA, USA) were used for the current study, but a new low-pressure protocol had to be developed. The standard Biolog inoculation protocol uses a semi-liquid inoculating fluid obtained from Biolog, Inc. that contains a gelling agent (phytagel) to stabilize the inoculating fluid in the wells. During preliminary experiments with the Biolog GN2 system that included the gelling agent, significant bubbling activity was observed in most wells at 0.7 kPa that likely was caused by the retention of dissolved gases at higher pressures in the inoculating fluid. The gelling agent appeared to retard the outgassing of the inoculating fluid, thereby causing the formation of unwanted bubbles overflowing individual wells. The bubbling rendered the Biolog GN2 assay plates ineffective at low pressures.

The first step in the low-pressure Biolog GN2 assay protocol was to replace the inoculating fluid with a non-gelling alternative. We tested 10 mM PO_4_ buffer, sterile deionized water (SDIW), and 0.5% NaCl (all at pH 7.2) as carrier fluids. The 10 mM PO_4_ buffer was chosen because it produced slightly higher colour development by the tetrazolium dye for several organics (Schuerger, data not shown).

Biolog GN2 plates were prepared by dispensing 100 µL of cell suspensions in 10 mM PO_4_ buffer into each well (approx. 2 × 10^5^ cells per well), adding 25 µL of SDIW to each well, and dispensing 150 µL of SDIW into the void spaces between wells in the Biolog GN2 plates. The addition of SDIW into void spaces between wells reduced the evaporation of water from the organics-containing wells at pressures between 0.7 and 5.0 kPa.

Replicates of GN2 plates were inserted into hypobaric desiccators, sealed, and maintained at 0.7 kPa for up to 49 d, as described below. At 0.7 kPa, the PO_4_ buffer in many wells would slowly evaporate, requiring the addition of 25–50 µL of SDIW (to maintain the osmotic strength and buffering capacity of the fluids) to each well as needed to return the volume of the incubating fluid to approx. 125 µL. The hypobaric desiccator at 0.7 kPa was vented twice weekly to examine and adjust the fluid levels in the wells.

And finally, negative controls were created without viable cells and co-incubated with inoculated GN2 plates for the duration of the experiments. When low pressure was not required, GN2-experimental and PO_4_-control plates were incubated in 2.5-L polycarbonate containers with gasket-sealed lids (AnaeroPak® System containers, Mitsubishi Gas Chemical, Co; obtained from Fisher Scientific).

Prior to quantitatively measuring the Biolog GN2 plate with a Genios Pro plate reader (Tecan USA, Research Triangle Park, NC, USA; Magellan v. 5.03 software), each well in the plate was stirred with sterile 10 µL pipette tips to resuspend the cells. The Genios system was set to measure the OD of wells in 10 spots within each well at 540 nm and 25 °C. The average of all 10 scan spots per well was automatically recorded and files converted to Excel CSV formatting for subsequent analysis.

### Hypobaric desiccators

The design and operation of the hypobaric chambers were described previously^5,14^. In brief, 4-L polycarbonate desiccators (model 08-642-7, Fisher Scientific) were connected individually to a low pressure controller (model PU-842, KNF Neuberger, Trenton, NJ, USA) in which the controller and pump were placed outside a -20 °C microbial incubator. The Biolog GN2 plates were inserted into the desiccators and their lids raised and rotated approx. 20° such that the lids were not seated as per normal operations, but instead were slightly raised above the tops of the wells eliminating cross-contamination of wells caused by the condensation of water vapour around the well lips. Four anaerobic pouches^61^ (model 23-246-378 AnaeroPak® System sachets, Fisher Scientific) were added around the stacked GN2 plates, followed by the addition of 150 mL of SDIW in a 200 mL beaker to increase the vapour pressure of water at 0.7 kPa. The desiccator tops were then placed upon silicone O-rings coated with vacuum grease (Dow Corning^TM^, obtained from Fisher Scientific) to enhance the sealing of the desiccators at 0 °C.

The desiccators were fitted with 0.22 µm air filters on both the vacuum lines and the vent lines (see Fig. 1)^5^ to reduce microbial contamination during pump operations. Prior to inserting the desiccators into the -20 °C microbial incubator, the desiccators were flushed for 3–4 min with ultrahigh purity (UHP) CO_2_ gas passed through the filtered vent lines.

### Experiment 1: Pressure-only effects between 2.5 and 101.3 kPa on carbon source utilization

To measure the effects of low pressure alone on the metabolism of individual carbon sources by *S. liquefaciens*, an experiment was designed to vary pressure between 2.5 and 101.3 kPa while holding gas composition at an Earth-normal 78% pN_2_ and 21% pO_2_ composition (lab atmosphere) and temperature (30 °C) constant. Due to high evaporation rates from the GN2 wells at 30 °C and 2.5 or 5.0 kPa, it was not possible to conduct assays at pressures below 2.5 kPa at 30 °C.

Biolog GN2 plates were prepared as described above, and inserted into separate hypobaric desiccators set at 2.5, 5.0, 10.0, 20.0, or 101.3 kPa. The GN2 plates were incubated for 48 h at 30 °C, the desiccators vented, the wells stirred, and the plates read with the Genios plate reader.

At 30 °C, the wells would lose approx. 1/3 of the starting volumes in each well. Thus, 40–50 µL of SDIW were added to each well, on an as needed basis, prior to reading the plates. Only two desiccators were available, and thus, hypobaric treatments were randomly assigned to each desiccator, and assays repeated until all five pressure treatments were conducted three times each with two GN2 replicates per assay (overall n = 6).

### Experiment 2: Effects of Mars versus Earth pressure, gas composition, and temperature on carbon source utilization

In order to probe the effects of low pressure at 0.7 kPa on carbon source utilization, both temperature and pressure had to be regulated simultaneously. Thus, the experimental design was altered and the following five treatments created:

1. Mars simulations in which GN2 plates were incubated at low pressure (0.7 kPa), low temperature (0 °C), and CO_2_-enriched anoxic atmosphere^61^ (i.e., low pO_2_; <1% pO_2_). These conditions are referred to as low-PTA conditions that simulate a portion of the Mars surface environment. All low-PTA assays were conducted in the 4-L desiccator systems described above. All other assays with GN2 plates were maintained in 2.5-L polycarbonate, gasket-sealed, containers from Fisher Scientific.
2. Earth Control-1 (EC-1) assays were incubated at 101.3 kPa, 0 °C, and a CO_2_-enriched anoxic atmosphere (see above).
3. Earth Control-2 (EC-2) assays were incubated at 101.3 kPa, 0 °C, and an Earth-normal atmosphere of 78:21 pN_2_ to pO_2_ conditions.
4. Earth Control-3 (EC-3) assays were incubated at 101.3 kPa, 30 °C, and a CO_2_-enriched anoxic atmosphere (see above).
5. Earth Control-4 (EC-4) assays were incubated at 101.3 kPa, 30 °C, and an Earth-normal atmosphere of 78:21 pN_2_ to pO_2_ conditions.

The low-PTA assays were vented twice weekly, SDIW added to wells as required, new anaerobic pouches placed into the desiccators, the desiccators flushed with UHP CO_2_ gas, and the conditions equilibrated to 0.7 kPa at 0 °C. The tetrazolium dye colour shifts expected from the GN2 wells following metabolic activity were very slow to develop at low pressure and low temperature, so the GN2 plates were only measured with the Genios plate reader after 35, 42, and 49 d from the start of the experiment. The EC-1 and EC-2 assays were read at the same time-steps as GN2 plates incubated at low-PTA conditions. In contrast, colour development in the EC-3 and EC-4 assays progressed quickly because they were incubated at 30 °C, and thus, the plates were read at 48 h.

At the end of the 49 d (i.e., low-PTA, EC-1, and EC-2 conditions) or 48 h (i.e., EC-3 and EC-4) assays, the GN2 plates were placed under lab-normal conditions at 101.3 kPa, 30 °C, and 78:21 pN_2_ and pO_2_ atmospheres to determine if the incubation conditions killed cells in negative wells. The assays were randomly assigned to the available desiccators and polycarbonate containers and the experiment conducted twice with two replicates per treatment per experimental run (overall n = 4).

### Data analysis

Absorbance values for each well containing a different carbon source were blanked against the values of the control well (i.e., the A1 well in the Biolog GN2 plates). All negative absorbance values were set to zero. A positive substrate response was taken to be an OD (at 540 nm) greater than 0.1 after the control well OD reading was subtracted. Total well colour development (TWCD) was calculated as the sum of the control well subtracted absorbance values of all 95 wells.

For principal component analysis (PCA) plots the data were normalized by dividing each absorbance value of a well by its average well colour development to minimize the influence of inoculum density difference as well as performance differences among plates^62^. The plots were created using the statistical environment RStudio^63^ and the default R package *stats* v3.4.4 applying the function *princomp* ().

All raw data for both experiments are provided in Supplementary Tables S1 (for Exp-1) and S2 (for Exp-2). The data are organized so that each replicate GN2 plate data forms a single column in each table.

## Electronic supplementary material

Dataset 1

Dataset 2
